# Structural Biology of STAT3 and Its Implications for Anticancer Therapies Development

**DOI:** 10.3390/ijms19061591

**Published:** 2018-05-28

**Authors:** Jacopo Sgrignani, Maura Garofalo, Milos Matkovic, Jessica Merulla, Carlo V. Catapano, Andrea Cavalli

**Affiliations:** 1 Institute for Research in Biomedicine (IRB), Università della Svizzera Italiana (USI), Via Vincenzo Vela 6, CH-6500 Bellinzona, Switzerland; jacopo.sgrignani@irb.usi.ch (J.S.); maura.garofalo@irb.usi.ch (M.G.); milos.matkovic@irb.usi.ch (M.M.); 2 University of Lausanne (UNIL), CH-1015 Lausanne, Switzerland; 3 Institute of Oncology Research (IOR), Università della Svizzera Italiana (USI), Via Vincenzo Vela 6, CH-6500 Bellinzona, Switzerland; jessica.merulla@ior.usi.ch (J.M.); carlo.catapano@ior.usi.ch (C.V.C.)

**Keywords:** STAT3, cancer, molecular modeling, drug design, structural biology

## Abstract

Transcription factors are proteins able to bind DNA and induce the transcription of specific genes. Consequently, they play a pivotal role in multiple cellular pathways and are frequently over-expressed or dysregulated in cancer. Here, we will focus on a specific “signal transducer and activator of transcription” (STAT3) factor that is involved in several pathologies, including cancer. For long time, the mechanism by which STAT3 exerts its cellular functions has been summarized by a three steps process: (1) Protein phosphorylation by specific kinases, (2) dimerization promoted by phosphorylation, (3) activation of gene expression by the phosphorylated dimer. Consequently, most of the inhibitors reported in literature aimed at blocking phosphorylation and dimerization. However, recent observations reopened the debate and the entire functional mechanism has been revisited stimulating the scientific community to pursue new inhibition strategies. In particular, the dimerization of the unphosphorylated species has been experimentally demonstrated and specific roles proposed also for these dimers. Despite difficulties in the expression and purification of the full length STAT3, structural biology investigations allowed the determination of atomistic structures of STAT3 dimers and several protein domains. Starting from this information, computational methods have been used both to improve the understanding of the STAT3 functional mechanism and to design new inhibitors to be used as anticancer drugs. In this review, we will focus on the contribution of structural biology to understand the roles of STAT3, to design new inhibitors and to suggest new strategies of pharmacological intervention.

## 1. Introduction

Transcription factors (i.e., DNA binding proteins controlling the rate of gene transcription; TFs) are nodal points in signaling pathways and among the most frequently affected genes in cancer [1,2]. Signal transducer and activator of transcription (STAT) is a family of cytoplasmic TFs (STAT1, STAT2, STAT3, STAT4, STAT5a, STAT5b, and STAT6) responsible for the transmission to the nucleus of signals from multiple receptors and non-receptor associated kinases [3,4].

In this review, we will focus on structural biology studies of a specific member of the STAT family, namely STAT3, and its implications for comprehension of the protein functional mechanism and the development of novel anticancer therapies. This protein plays a pivotal role in the regulation of several genes involved in proliferation, differentiation, apoptosis, angiogenesis and immuno-inflammatory processes. Additionally, it has been recently demonstrated that STAT3 also localizes into mitochondria and that it can influence their function [5,6,7].

Over the years, enhanced and dysregulated STAT3 activity has been observed in a large number of cancer cell lines [8], indicating this protein as a promising target for the development of anticancer therapies [9,10,11,12]. In fact, STAT3 drives the expression of proliferation and survival genes, like *c-myc*, *bcl-XL*, *mcl-1* [3,13,14]. Furthermore, it has important consequences on the tumor microenvironment by increasing the expression of pro-angiogenic factors [3,15]. Finally, STAT3 activation in tumors induces immune-suppressive cytokines and promotes immune-evasion [16,17,18].

A search in the Web of Science database for papers with the word “STAT3” and “inhibitor” in the title revealed more than 500 articles published in the last twenty years. This data makes explicit the great effort made by the scientific community to develop pharmacological therapies based on the modulation of STAT3 functions.

**Figure 1:**
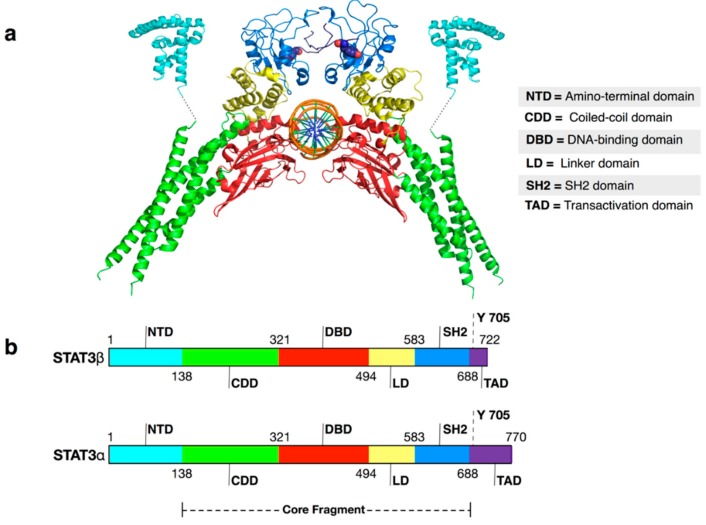
(**a**) Cartoon representation of USTAT3β: DNA structure (PDB ID 4ZIA for the N-termini and 4E68 for the remaining structure). Color keys: cyan = amino-terminal domain; green = coiled-coil domain; red = DNA-binding domain; yellow = linker domain; blue = SH2 domain; violet = transactivation domain; orange = DNA. Tyrosine 705 residues are shown as spheres. In the lower part of the picture, a scheme of STATs domain division is reported; (**b**) Schemes of STAT3α and STAT3β domain division. The dashed line represents the core fragment of the STATs domain (inspired by a scheme presented by Chen et al. [26] for STAT1).

Despite the significant efforts made, the tendency of the STAT3 to aggregate prevented, until now, the determination of the structure of the entire protein in both monomeric and dimeric form. However, several recombinant proteins not prone to aggregation have been expressed and their structure solved by X-ray crystallography (Table 1). These investigations [19,20,21] confirmed that STAT3 shares with other members of the STAT family a peculiar 3D-structure characterized by six main structural motifs (Figure 1): (1) Amino-terminal domain (NTD), (2) coiled-coil domain, (3) DNA-binding domain, (4) linker domain (LD), (5) Src Homology 2 (SH2) domain and (6) trans-activation domain (TAD). The domain at the C-terminal of STAT3, TAD, is intrinsically disordered and highly conserved between STAT proteins. Several experiments indicate that the TAD is not involved directly in dimerization interface of many STATs proteins. However, when phosphorylated, a specific tyrosine residue (Tyr705 in the case of STAT3) included in the TAD can reinforce the protein-protein interaction binding in a specific site located in the other protein partner [22,23,24,25].

Different splicing results in two main STAT3 isoforms (α and β) that differ for the length of the TAD (∼50 residues in STAT3α and ∼7 residues in STAT3β). The biological roles of the two isoforms have been debated since their discovery. However, because the high disorder that marks this protein region it has been scarcely characterized from the structural point of view. Therefore, in this review we will only discuss the other structured domains that are common in both isoforms.

## 2. Functional Mechanism

Cytokine receptors and growth factor receptors are the main drivers of STAT3 activation. Moreover, it has been shown that also environmental factors such as smoking cigarettes, infections and stress can lead to STAT3 triggering by toll-like receptors (TLR), adrenergic receptors and nicotinic receptors [16].

The interaction of the physiological ligands with their receptors starts the so-called “canonical” STAT3 activation pathway that involves phosphorylation of a specific tyrosine residue (Tyr705) in the TAD [3,16]. This post-transcriptional modification is mainly catalyzed by a family of receptor-associated tyrosine kinases, JAK1–JAK3 and Tyk2 [28], but also by non-receptor kinases like c-src and c-abl [3].

Phosphorylation changes the propensity of STAT3 molecules to form homo-dimers [3]. Similar to the STAT1 dimer-DNA structure [26,29], while the main dimerization interface is formed by the SH2 domains, the two TADs increase the stability of the dimer by the binding of p-Tyr705 in a specific cavity located in the SH2 domain of the other monomer. A key element for the p-Tyr705 recognition is the presence of an arginine, strictly conserved in all known SH2 domain (Arg609 in STAT3) residing in the interior of the SH2 domain [26]. This arginine residue, because of the favorable electrostatic interaction energy between the negatively charged phosphate and the positive NH_3_ amino group, stabilizes the p-Tyr705 binding. Phosphorylated STAT3 is retained in the nucleus, where it binds to DNA in order to promote and activate transcription of a wide array of genes controlling cell differentiation, proliferation and survival in various cell types.

Year after year, is becoming clearer that the phosphorylation–dimerization–activation scheme does not completely recapitulate the complexity of the STAT3 functional mechanisms and a novel more complicated picture, involving unphosphorylated STAT3 and other post-transcriptional modifications, is emerging [30]. For example, the presence of unphosphorylated STAT proteins (USTATs) inside the nucleus, as well as, their ability to dimerize and bind DNA have been shown experimentally [20,31,32,33,34]. Concerning their functional roles, the formation of USTAT3 dimers influences the nuclear localization of the protein, DNA-binding, chromatin-remodeling and the regulation of specific gene expression [27].

The NTD domain is not directly involved in the formation of phosphorylated STAT3 dimers or in their interaction with DNA, therefore it was not considered central for the STAT3 functions for a long time. However, recent studies indicated that NTD has an important role at low cytokine concentration, i.e., when the activated STAT3 concentration is small. In fact, the NTD domains seem to facilitate the STAT3 binding to weak STAT3-binding sites by forming tetramers composed of a pair of phosphorylated dimers [27]. Moreover, both experimental [35] and computational studies [25,36] indicated that the NTD is part of the dimerization surface in USTAT3 dimers and that it is important for nuclear accumulation [37], DNA binding [31] and gene expression regulation [38]. Collectively, these studies revealed that also this protein region could be suitable for the development of new drugs, however, few attempts were done [39,40] and a more intense research activity is required to develop inhibition strategies based on NTD binding.

## 3. Post-Transcriptional Modifications and Their Role in the STAT3 Function

Phosphorylation of tyrosine residues located in the SH2 and TAD domains of STAT proteins by protein tyrosine kinases (PTKs) has been identified as a critical event for modulating their functions [24,41]. However, other post-transcriptional modifications (PTMs) can influence the protein activity.

For example, STAT3 is subject to phosphorylation on serine 727 (that lacks in STAT3β) by serine protein kinases, as well as, lysine acetylation and methylation by protein acetyltransferase and methyltransferases [16,42]. Multiple studies reported about the possible roles of Ser727 phosphorylation in both protein functions and progression of specific cancers [43]. Moreover, Wakahara et al., reported that Ser727 phosphorylation regulates the duration of the STAT3 transcriptional activity by bolstering the p-Tyr705 dephosphorylation [44].

Concerning acetylation, recent studies supported the hypothesis that it modulates different functions and properties of STAT3 [45] such as protein dimerization [46], transcriptional activity [47], mitochondrial translocation [48], cardiogenesis [49] and methylation of tumor-suppressor gene promoters [50]. Other studies lead to the identification of additional acetylation sites: K685, K49, K87, K679, K707, K709 [45].

Regarding methylation, Lee and coworkers recently demonstrated that Enhancer of Zest Homolog 2 (EZH2), a histone-lysine N-methyltransferase, interacts with and methylates STAT3 increasing its activity [51].

Finally, in 2014 Mariotto and coworkers reported that the S-Glutathionylation of Cys328 and Cys542, located in the DNA-binding domain and in the linker domain, respectively, severely impair STAT3 phosphorylation [52].

## 4. Structural and Biophysical Investigations

As already pointed out, structural studies on STAT3 and other STAT proteins, have been slowed down by problems with protein expression and purification due to the tendency of STATs to form aggregates. As consequence, only domains of the STAT proteins have been solved and the structure of full length STAT3 is not available in the protein data bank (PDB).

The first structure (PDB code 1BG1) of mouse phosphorylated STAT3β core fragment (i.e., lacking NTD) in complex with DNA (see Figure 1) was solved in 1998 by Becker and coworkers with a resolution of 2.25 Å [21]. This structure enabled a detailed characterization of the interaction between DNA and the STAT3-DNA binding domain and the identification of residues essential for the interaction. Moreover, the binding of p-Tyr705 to SH2 was characterized in detail. In particular, the structural studies confirmed that p-Tyr705 forms polar interactions with residues Lys591, Arg609, Ser611, and Ser613. This information has been crucial for drug design given that the majority of STAT3 inhibitors reported in the literature so far has been designed to compete with p-Tyr705 for the binding in its SH2 cavity.

In 2008 Chen and coworkers solved by X-ray crystallography the structure of the monomeric core fragment of USTAT3 (PDB code 3CWG) at a resolution of 3.05 Å [19]. This study confirmed that STAT3 and USTAT3 have essentially an identical structure and indicated that the core fragment is primarily monomeric.

The same core fragment, with the NTD replaced by green fluorescent protein (GFP), was solved in complex with DNA (PDB code 3E68) at a resolution of 2.65 Å by Nkansah et al., in 2013 [20]. Notably, in this case, it was possible to observe for the first time the complex formed by two USTAT3 molecules and a DNA fragment, demonstrating that Tyr705 phosphorylation is not the only event triggering STAT3 dependent transcription.

Finally, in 2015 the first structure of the NTD was published by Frank and coworkers [27]. During their experiment, the authors found that the removal of the first two residues (Met1 and Ala2) from the protein sequence significantly increased the protein solubility. In particular, they expressed different constructs (residues 3 to 120, 3 to 124, 3 to 126, 3 to 130, 3 to 135, and 3 to 138), but only the one with residues from 2 to 138 produced crystals. The obtained 3D structure of STAT3-NTD monomers is very similar to those of STAT1 [53] and STAT4 [54].

**Figure 2:**
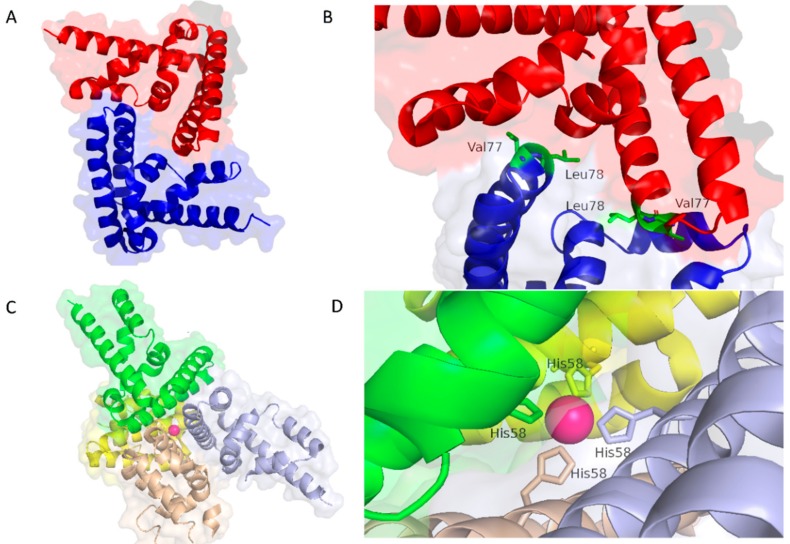
(**A**) NTD handshake dimerization mode and (**B**) details about the interactions stabilizing this configuration. The two NTDs are shown in blue and red cartoons and the residues important for the dimerization in green sticks. (**C**) Ni2^+^ mediated multimerization (in this case the different NTDs are depicted in green, violet, yellow and sandy and (**D**) details about the Ni2^+^ coordination. Structures extracted from the PDB entry 4ZIA [27].

Concerning the interaction between multiple NTD, the asymmetric unit of the crystal is formed by five NTDs and two different protein-protein interaction surfaces were observed, one indicated as ‘“handshake” interface and the other as “Ni2^+^-mediated” (Figure 2). It is important to note that, while the first interaction mode is accepted to be present in physiological conditions, the authors state that “Ni2^+^-mediated” interaction is probably due to the purification conditions. However, considering (1) the tendency of STAT3 to form paracrystals [23,55] or nuclear bodies to defend itself from phosphorylation and (2) that its function is directly influenced by metals [56], also the “Ni2^+^-mediated” interface could be necessary for some physiological, still not well characterized, effects.

Interestingly, in the previously discussed study [27], the NTD dimerization was also investigated by SEC—small-angle X-ray scattering (SEC-SAXS) experiment. The obtained results confirmed not only the NTD dimerization, but also that this methodology is a very useful tool to study dynamics and aggregation prone samples.

Computational studies also have been carried out to investigate the structure of STAT3 monomer and dimer. In 2012, Husby et al. [57] investigated the interaction between STAT3, USTAT3 and DNA by molecular dynamics simulations obtaining interesting data about the importance of specific residues in the interaction with DNA.

More recently, some of us used protein-protein docking and molecular dynamics simulations to investigate the structure of USTAT3 dimers not bound to DNA [25]. In particular, the models obtained by docking were screened by the available structural information and then molecular dynamics simulations were used to relax the models. Finally, Molecular Mechanics-Generalized Born Surface Area (MM-GBSA) [58] calculations have been used to identify the residues more important for the dimer stability.

## 5. Drug Design

Over the years, experimental information about the structure of STAT3 and computer simulations have been used to (1) identify new inhibitors, (2) to better understand the binding mode of existing inhibitors and (3) to improve their affinity for the target. A list of papers published in the last five years, together with a summary, is reported in Table 2. Multiple strategies, involving different protein regions, have been followed aiming to design potent and selective inhibitors of the STAT3 functions [59,60,61,62].

The goal of this section is to give an overview about how structural biology influenced the discovery of new biologically active molecules. The medicinal chemistry efforts to discover STAT3 inhibitors have been extensively reviewed elsewhere [9,11,63,64,65,66]. Here, therefore, we will discuss only selected examples of ligand-based drug design and how computational, structural and biochemical techniques have been used to understand the binding mode of STAT3 inhibitors. The majority of the ligands have been designed to antagonize the phosphorylation induced protein dimerization. In this case, the rationale that guided the screening and the structural optimization of the lead compounds was the identification of molecules able to compete with p-Tyr705 for binding to a site located in the SH2 domain [67].

**Figure 3:**
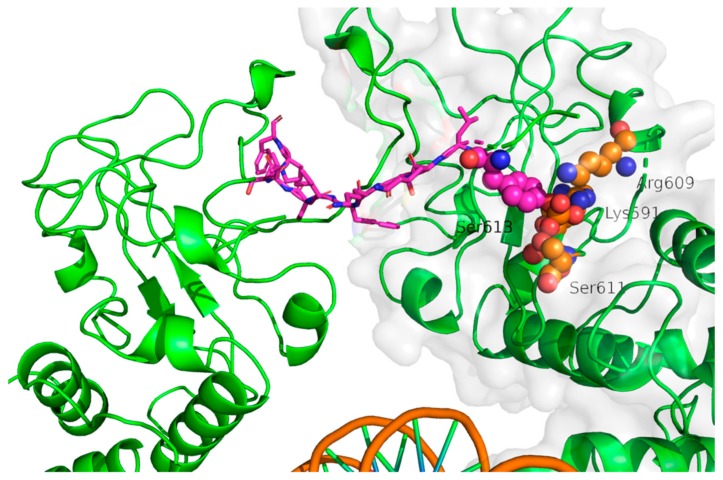
Insight on the Y*LKTKF (represented in sticks, carbon atoms colored in magenta) interaction in a STAT3 dimer (represented as green cartoons, PDB code 1BG1 [21]). Important residues in contact with Y* are depicted as spheres (carbon atoms colored in orange).

Among the STAT3 inhibitors designed to compete for p-Tyr705 binding site a large group is formed by phosphopeptides. This approach was firstly attempted by Turkson et al. in 2001 [68]. Starting from the sequence of the SH2 binding peptide (PY*LKTK, where Y* indicates p-Tyr705) they carried out a systematic analysis of the binding properties of derived peptides. After an in-depth characterization of the in vitro and in vivo activity of PY*LKTK and the other synthesized peptides, they concluded that the sequence XY*L (X is a generic residue) represents the minimal active sequence. Using a similar approach McMurray and coworkers [69] considered tyrosine-phosphorylated hexapeptides, selected taking into consideration the STAT3 docking sites for gp130, LIFR, EGFR, IL-10R, and GCSFR. They discovered one peptide (sequence Y*LPQTV) able to block STAT3 dimerization and DNA binding with an IC_50_ of 150 nM (determined by electrophoretic mobility shift assay, EMSA) [69] or 290 nM (determined by fluorescence polarization, FP) [70]. The subsequent optimization of this peptide benefitted of the structural information about the interaction between one STAT3 monomer and the Y*LKTKF peptide from the other STAT3 molecule forming the “canonical” dimer reported in the seminal work of Becker et al. [21] (PDB code 1BG1, Figure 3). However, the X-ray structure did not give any information about the interaction of the PQTV portion of the lead peptide, because this is not present in the co-crystallized peptide. Then, structure-activity relationship (SAR) analyses were performed to identify residues that are important for binding and could be modified. Collectively, these studies led to the identification of a peptidomimetic (hydroxycinnamoyl-Tyr(PO3H2)-Leu-*cis*-3,4-methanoPro-Gln-NHBn) that showed an IC_50_ of 150 nM in FP assays [70]. More recently, the same group published a peptidomimetic inspired by their best phosphopeptide that displayed an IC_50_ 162 nM [71]. Structure-based computational methods have been applied to identify many small organic compounds able to modulate STAT3 activity by binding to the SH2 domain. For example, the structural and computational analysis of the interaction between phosphotyrosine peptides and STAT3 guided the development of a peptidomimetic molecule (S3I-M2001) [72]. The list of the molecules discovered by virtual screening includes STAT-21 [73], STX-0119 [74] and a group of three molecules named cpd3, cpd30, and cpd188 that compete with Y* for its binding site in the SH2 domain [75].

Computational methods, based on the use of the available structural information, have also been used to improve our knowledge of how already identified compounds interact with STAT3. For example, Brambilla et al. [76] used an integrated approach between computational and experimental methods to identify the binding site for OPB-31121, a STAT3 inhibitor discovered by biochemical/cellular assays. In particular, computational studies based on docking, molecular dynamics and free energy calculations suggested that OPB-31121 binds to a site different from that of other characterized STAT3 inhibitors, such as S3I.201. Free energy calculations gave essential suggestions about the residues more critical for the binding of OPB-31121 to the SH2 domain. Then, two mutants (S636A and V637A) were expressed and their ability to bind OPB-31121 and S3I.201 was tested by isothermal titration calorimetry (ITC) experiments. Interestingly, these mutations abrogated only the binding of OPB-31121 and not of S3I.201, confirming that computer simulations correctly identified the specific OPB-31121 binding site. This strategy was used by the same group to investigate the binding of another structurally similar STAT3 inhibitor, OPB-51602 [5].

Importantly, also other STAT3 domains have been explored to design innovative inhibitors. The relevance of the NTD in USTAT3 dimerization, oligomerization [66] and other cellular processes motivated Timofeeva at al. [40] to design peptides able to bind this region and inhibit specific STAT3 functions. Starting from the analysis of the STAT4 NTD dimers structure, available at the time of their study (PDB code 1BGF [54]), they selected two helices of 12 and 20 residues involved in the binding surface. Then, they synthetized peptides considering the sequence the corresponding to the same protein region in STAT3 and hypothesizing that they might antagonize the NTD dimer formation.

**Figure 4:**
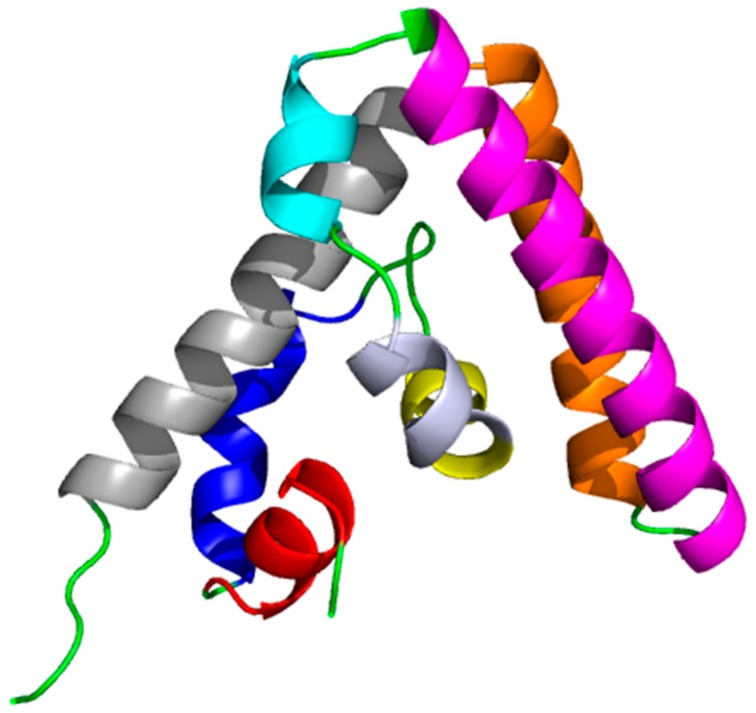
STAT4-NTD domain (PDB code 1BGF [54]), alpha-helixes are color coded 1-red, 2-blue, 3-yellow, 4-bluewhite, 5-cyan, 6-magenta, 7 orange, 8 gray.

The complex between the two peptides and the STAT4 NTD was investigated by nuclear magnetic resonance (NMR). The structure with the 20-residues long peptide was not determined because it induced protein aggregation, probably due to NTD unfolding. Also, the experiments on the 12-residues long peptide, analog of helix 2 (Figure 4), suggested an effect on the NTD structure. Nevertheless, in this case, it was possible to carry on the analysis and observe that the majority of the chemical shift changes were located in the region occupied by helix 8. The authors concluded that a new interaction interface, not detected in the NDT STAT4 structure [54], was induced by the presence of the 12-residues peptide.

Finally, starting from the helix 2 analog, a library of mutant peptides was synthetized and their ability to suppress STAT3 signaling in cancer cells was evaluated. To note, these peptides were fused with penetratin, a peptide able to permit the entrance in the cells of non-cell penetrating molecules [77].

Some of the synthetized peptides demonstrated STAT3 inhibitory activity in the gamma activation sequence (GAS)-luciferase reporter assays and inhibition of proliferation of Michigan Cancer Foundation (MCF)-7 breast cancer cells, confirming that the NTD domain is a protein region suitable for the development of new anticancer drugs.

In summary, these studies provide clear evidence of the crucial role of structural biology information for the identification and optimization of STAT3 inhibitors and indicate that future studies cannot disregard the use of computational and experimental structural techniques.

## 6. Conclusions

In summary, despite difficulties in the expression and crystallization of the protein, structural biology investigations have been of great help in improving our understanding of the STAT3 structure and its functional mechanism.

The structures available in the PDB have been the starting point for a large number of studies in which computer simulations have been used to identify new drugs, improving their affinity for the target or simply to understand their biding mode.

However, a lot of issues, in particular concerning the structure of USTAT3 dimers, the interaction with specific drugs but also the role of NTD domains in the phosphorylated dimers and other molecular events, are still open and should be the subjects of new studies.

Hopefully, in the next future the integration between computational, structural and biophysical techniques will help to better characterize from the structural point of view the drug protein complexes and those STAT3 species that have eluded the attempts of characterization carried out so far.

## Figures and Tables

**Table 1:** STAT3 structures available in the protein data bank (PDB).

PDB Code	Description	Reference
3CWG	Unphosphorylated mouse STAT3 core fragment (full length without amino-terminal domain (NTD))	[19]
1BG1	STAT3B/DNA complex (no N-terminal domain)	[21]
4E68	Unphosphorylated STAT3B (no N-terminal domain) core protein binding to dsDNA	[20]
4ZIA	X-ray structure of STAT3 N-terminal domain	[27]

**Table 2:** Summary of more relevant papers, published in the last five years, in which structural biology and computational methods were used to design and/or to better understand the molecular determinants of STAT3 inhibitors.

Title	Structural Experimental and/or Computational Structural Biology Contribution	Year	Reference
*Arctigenin Inhibits STAT3 and exhibits anticancer potential in human triple-negative breast cancer therapy.*	Use of docking and molecular dynamics simulations to understand the binding mode of Arctigenin (a STAT3 inhibitor).	2017	[78]
*Mitochondrial dysfunction induced by a SH2 domain-targeting STAT3 inhibitor leads to metabolic synthetic lethality in cancer cells*	Use of docking and molecular dynamics simulations to characterize the binding mode of OPB-51602, a small molecule currently in clinical trials, to STAT3.	2017	[5]
*Discovery of an Orally Selective Inhibitor of Signal Transducer and Activator of Transcription 3 Using Advanced Multiple Ligand Simultaneous Docking*	Use of Advanced Multiple Ligand Simultaneous Docking (AMLSD) to design compounds able to directly inhibit both phosphorylation and dimerization of STAT3 protein.	2017	[79]
*Identification of New Shikonin Derivatives as STAT3 Inhibitors*	Discovery of to discover PMMB-187, a new STAT3 inhibitor by modification of shikonin scaffold guided by computational modeling.	2017	[80]
*Identification of New Shikonin Derivatives as Antitumor Agents Targeting STAT3 SH2 Domain.*	Discovery of PMM-172, a new STAT3 inhibitor, via molecular docking and scaffold modification of shikonin.	2017	[81]
*MMPP Attenuates Non-Small Cell Lung Cancer Growth by Inhibiting the STAT3 DNA-Binding Activity via Direct Binding to the STAT3 DNA-Binding Domain*	Use of molecular docking for the rational design of 16BHPB, a STAT3 inhibitor.	2017	[82]
*Identification of novel small molecules that inhibit STAT3-dependent transcription and function*	Use of docking studies to understand the binding mode of new STAT3 inhibitors identified by high-throughput screening.	2017	[83]
*Small molecules inhibit STAT3 activation, autophagy, and cancer cell anchorage-independent growth*	Molecular docking of STAT3 inhibitors to predict their binding mode.	2017	[84]
*Discovery of monocarbonyl curcumin-BTP hybrids as STAT3 inhibitors for drug-sensitive and drug-resistant breast cancer therapy*	Docking studies to predict the binding mode of new STAT3 inhibitors.	2017	[85]
*Applying Small Molecule Signal Transducer and Activator of Trancription-3 (STAT3) Protein Inhibitors as Pancreatic Cancer Therapeutics*	Docking of STAT3 inhibitors and visualization of the computed receptor-ligand complexes.	2016	[86]
*4-Carbonyl-2,6-dibenzylidenecyclohexanone derivatives as small molecule inhibitors of STAT3 signaling pathway*	Docking studies of a new STAT3 inhibitor to confirm its binding mode.	2016	[87]
*Design, synthesis and evaluation of XZH-5 analogues as STAT3 inhibitors*	Structure activity relationship (SAR) to design new STAT3 inhibitors using docking studies to predict their binding mode.	2016	[88]
*Design, synthesis and biological evaluation of benzyloxyphenyl- methylaminophenol derivatives as STAT3 signaling pathway inhibitors*	SAR studies of benzyloxyphenyl-methylaminophenol scaffold and docking studies to confirm inhibitors binding mode	2016	[89]
*A novel small molecule STAT3 inhibitor, LY5, inhibits cell viability, colony formation, and migration of colon and liver cancer cells*	Evaluation of LY5 inhibition and docking studies to understand its binding mode	2016	[90]
*Screening and biological evaluation of a novel STAT3 signaling pathway inhibitor against cancer*	Identification of new STAT3 inhibitors (directed to SH2 domain) by docking studies using SPECS libraries (202,490 compounds).	2016	[91]
*Selective Inhibition of STAT3 with Respect to STAT1: Insights from Molecular Dynamics and Ensemble Docking Simulations*	Molecular mechanisms studies about the selectivity of 13 experimentally tested STAT3 inhibitors by molecular dynamics and ensemble docking simulations.	2016	[92]
*Antagonizing STAT3 activation with benzo[b]thiophene 1, 1-dioxide based small molecules*	Discovery of new STAT3 inhibitors via structure-based drug design methods, using benzo[b]thiophene 1, 1-dioxide (BTP) as lead compound.	2016	[93]
*Alantolactone selectively suppresses STAT3 activation and exhibits potent anticancer activity in MDA-MB-231 cells*	Structure-based molecular docking study used to investigate the binding mode of alantolactone, found to be a STAT3 inhibitor, and STAT3	2015	[94]
*Identification of Lead Compounds as Inhibitors of STAT3: Design, Synthesis and Bioactivity*	Virtual screening to find new STAT3 inhibitors.	2015	[95]
*Eriocalyxin B Inhibits STAT3 Signaling by Covalently Targeting STAT3 and Blocking Phosphorylation and Activation of STAT3*	Computational modeling analysis of the mechanism of action EB, a natural compound able to act as a STAT3 inhibitor.	2015	[96]
*Hitting the right spot: Mechanism of action of OPB-31121, a novel and potent inhibitor of the Signal Transducer and Activator of Transcription 3 (STAT3)*	In silico studies to understand the binding mode of a previously known STAT3 inhibitor.	2015	[76]
*Identification of STAT1 and STAT3 Specific Inhibitors Using Comparative Virtual Screening and Docking Validation*	Virtual screening to find new STAT3 inhibitors.	2015	[97]
*Discovery of a small-molecule inhibitor of STAT3 by ligand-based pharmacophore screening*	Pharmacophore based virtual screening.	2014	[98]
*A novel inhibitor of STAT3 homodimerization selectively suppresses STAT3 activity and malignant transformation*	Structure activity relationship (SAR) studies using a previously published S3I-201 compound as a lead and docking studies to understand the binding mode of the new inhibitors	2013	[99]
*Binding Modes of Peptidomimetics Designed to Inhibit STAT3*	Molecular docking combined with molecular dynamic simulations to model to model the structures of 12 peptidomimetic inhibitors bound to the SH2 domain of STAT3.	2012	[100]

## Abbreviations

NTD	N-terminal domain
STAT3	Signal transducer and activator of transcription 3
USTAT3	Unphosphorylated STAT3
TAD	Trans activation domain
LD	Linker domain
DBD	DNA binding domain
CDD	Coiled coil domain
NTD	N-terminal domain
SH2	SH2 domain
p-Tyr705	Phosphorylated tyrosine 705
PTMs	Post-transcriptional modifications
